# Bionic Integrated Positioning Mechanism Based on Bioinspired Polarization Compass and Inertial Navigation System

**DOI:** 10.3390/s21041055

**Published:** 2021-02-04

**Authors:** Qingyun Zhang, Jian Yang, Panpan Huang, Xin Liu, Shanpeng Wang, Lei Guo

**Affiliations:** 1 School of Automation Science and Electrical Engineering, Beihang University, Beijing 100191, China; qyzhang@buaa.edu.cn (Q.Z.); xliubuaa@buaa.edu.cn (X.L.); lguo@buaa.edu.cn (L.G.); 2 Beijing Advanced Innovation Center for Big Data-Based Precision Medicine, School of Medicine and Engineering, Beihang University, Beijing 100191, China; 3 Hangzhou Innovation Institute, Beihang University, Hangzhou 310051, China; z5042207@zmail.unsw.edu.au; 4 School of Instrumentation and Optoelectronic Engineering, Beihang University, Beijing 100191, China; wangshanpeng@buaa.edu.cn

**Keywords:** autonomous positioning, polarized skylight, inertial navigation, integrated navigation

## Abstract

In this paper, to address the problem of positioning accumulative errors of the inertial navigation system (INS), a bionic autonomous positioning mechanism integrating INS with a bioinspired polarization compass is proposed. In addition, the bioinspired positioning system hardware and the integration model are also presented. Concerned with the technical issue of the accuracy and environmental adaptability of the integrated positioning system, the sun elevation calculating method based on the degree of polarization (DoP) and direction of polarization (E-vector) is presented. Moreover, to compensate for the latitude and longitude errors of INS, the bioinspired positioning system model combining the polarization compass and INS is established. Finally, the positioning performance of the proposed bioinspired positioning system model was validated via outdoor experiments. The results indicate that the proposed system can compensate for the position errors of INS with satisfactory performance.

## 1. Introduction

Real-time autonomous positioning is essential for long endurance vehicles, especially in unknown and complex environments [1]. Currently, the global navigation satellite system (GNSS), visual navigation system (VNS), and inertial navigation system (INS) are the commonly used positioning methods [2,3]. However, the GNSS positioning performance could deteriorate when GNSS signals suffer from electromagnetic interference. VNS relies on useful information derived from the structured environment [4,5]. Currently, it is important to develop a global positioning system, which is not dependent on GNSS and VNS for long-range navigation.

In recent decades, researchers have found that many animals are able to perceive polarized skylight distributed across the sky for orientation [6,7,8]. Migrant songbirds can even use the polarized skylight cue to calibrate other compass systems when they migrate from one breeding site to another with voyages of up to thousands of kilometers [9]. In recent years, the polarization pattern has been proven to contain the geographic location information, which can be used for long-endurance vehicle positioning in unknown environments [10]. Due to the non-accumulative errors and immunity to electromagnetic interference, the bioinspired polarization positioning method has attracted much attention in the field of autonomous navigation.

Inspired by the polarization navigation mechanism of animals, the attitude determination methods based on the polarized light compass have been proposed [11,12,13,14,15]. To address the challenge of vehicle positioning based on the polarization pattern, Mei et al. proposed a bioinspired positioning method based on the polarized skylight [16]. By combining with the polarized skylight and the horizontal heading information provided by an electronic compass, the geographical position (latitude and longitude) of users could be deduced by the measurement of directions of polarization (E-vector) at two independent observations. Inspired by birds’ and Vikings’ ancient navigational skills [17], Chu et al. proposed a novel real-time positioning method based on the polarized skylight and geomagnetic field [18]. The positioning system consists of two polarized skylight sensors, a magnetic compass, and a level measuring instrument. The sun elevation is obtained based on the E-vector and the horizontal attitude, and the sun azimuth is calculated using the E-vector and the geomagnetic field. Then, the geographical position can be deduced when combined with the solar ephemeris. To improve the robustness and accuracy of polarization positioning under complex environments, in which the polarization pattern is weak and locally destroyed, Liu et al. employed a pulse coupled neural network to enhance the polarization positioning performance. It has been shown that the proposed method outperforms the aforementioned ones [19]. Considering the underwater environment, a bio-inspired polarization-sensitive imager to determine the position based on radial underwater polarization patterns is designed [20], the 61 km average accuracy for underwater positioning can be achieved. Subsequently, to improve the polarization positioning accuracy in magnetic disturbance conditions, Yang et al. have proposed a global positioning system based on the degree of polarization (DoP) pattern and the sun elevation difference algorithm to deduce the position information [21]. However, the accuracy of the calculated sun elevation based on the DoP pattern depends on the sky conditions [22,23]. In addition, the sun elevation difference positioning algorithm is based on two independent observations at different instants, which could result in a delayed positioning calculation. Hence, considering the limitations in the above literature, how to make full use of the polarization information to improve the sun elevation calculation accuracy and how to achieve real-time autonomous positioning of vehicles in an electromagnetic interference environment are fundamental difficulties in the field of polarization positioning.

With the identified difficulties in mind, this paper aims to propose a bionic integrated positioning mechanism combined with a polarized skylight and INS, and establish a bionic integrated positioning system model. The main contributions of this paper can be summarized as follows: A bionic integrated positioning algorithm combined with a polarized skylight and INS is proposed, and the bionic integrated positioning system model is established. The proposed model can provide real-time autonomous positioning in the presence of electromagnetic interference. In addition, a sun elevation calculation method based on DoP and E-vector is presented. The method can make full use of the polarization information across the sky and the accuracy and environmental adaptability of the proposed model can thus be improved.

The remainder of this paper is organized as follows. First, the integrated system design of the strap-down inertial navigation system (SINS) and the polarization navigation system (PNS) is introduced in Section 2. The integrated system model is presented in Section 3. To evaluate the positioning performance of the integrated system with the proposed model, the results of an outdoor experiment are analyzed in Section 4. The factors that affect the calculation accuracy of sun elevation are discussed in Section 5. Finally, some conclusions are drawn in the last section.

## 2. The SINS/PNS Integrated System Design

### 2.1. Coordinate Systems and Notations

**Figure 1:**
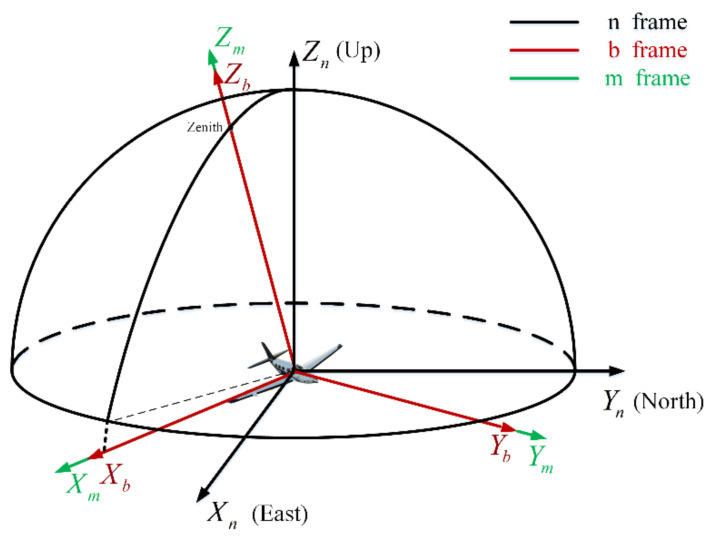
The relationship of the coordinate frame.

For the convenience of comprehension, it is necessary to define each coordinate frame used in this paper. The coordinate frames are defined as follows (Figure 1):

The n-frame: The navigation coordinate frame selects the geographic coordinate frame (E-N-U coordinate frame). The origin is the vehicle’s center of gravity. The $X_{n}$-axis, $Y_{n}$-axis, and $Z_{n}$-axis point towards the geography of East, North, and Upward to the Earth’s surface.

The b-frame: The body coordinate frame is the frame fixed on the vehicle. The origin is the vehicle’s center of gravity. The $X_{b}$-axis points right along the vehicle’s horizontal axis. The $Y_{b}$-axis points forward along its longitudinal axis, and the $Z_{b}$-axis points up along its vertical axis.

**Figure 2:**
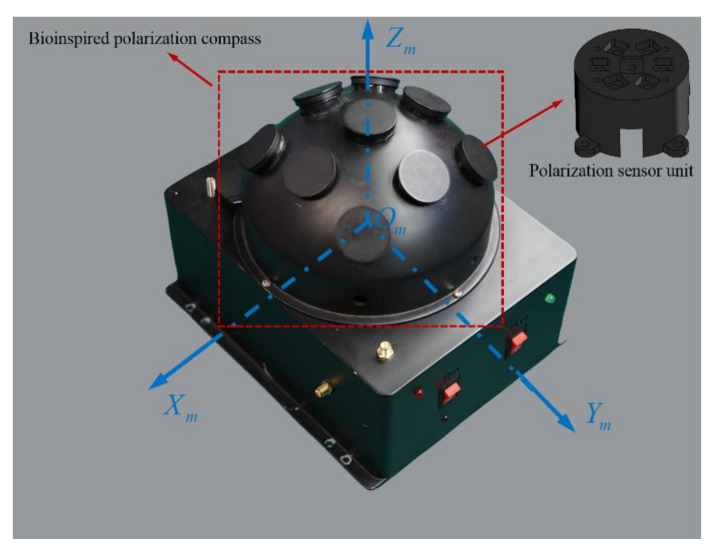
The structure of the bioinspired polarization compass.

The m-frame: The bioinspired polarization compass coordinate frame is defined as the mounting position of the polarization sensor units relative to the body frame. Its origin is at the center of the bioinspired polarization compass. The $Z_{m}$-axis is aligned with the observation direction of the middle polarization sensor units. The $X_{m}$-axis points to the zero-position direction of the middle polarization sensor units. The $Y_{m}$-axis satisfies the righthand rule (Figure 2).

### 2.2. The Polarization Information Detection System

To calculate the sun elevation based on the polarization pattern in the sky, the polarized skylight information (DoP and E-vector) should be obtained first. The bioinspired polarization compass used in this paper is of a hemispherical structure, which includes nine polarization sensor units. The polarization sensor units are distributed in an array on the surface of the hemisphere, and each polarization sensor unit is an independent observation point. The coordinate transformation relationship of each polarization sensor unit is determined by the compass structure. The structure of the compass is shown in Figure 2. Based on the bioinspired polarization compass, the polarized skylight information in different directions can be observed even when part of the sky is blocked.

### 2.3. The Hardware Design for the SINS/PNS Integrated System

**Figure 3:**
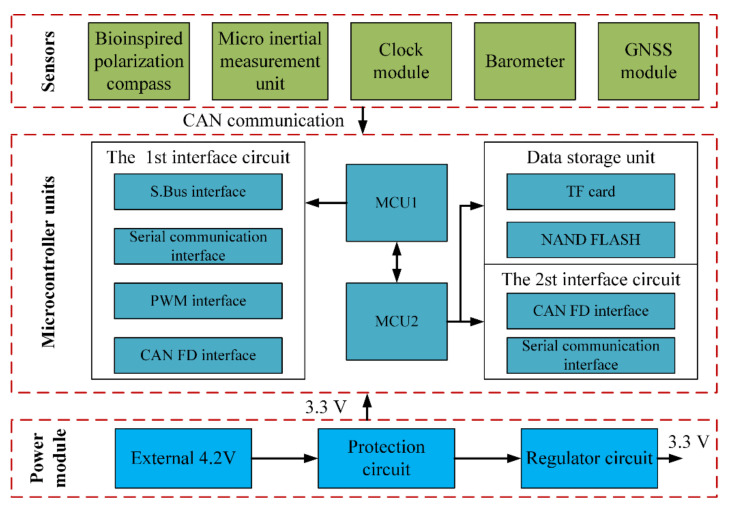
The strap-down inertial navigation system/polarization navigation system (SINS/PNS) integrated system block diagram.

**Figure 4:**
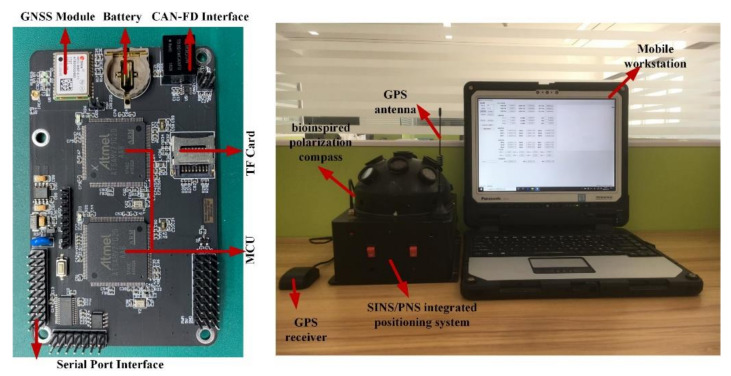
The hardware structure of the SINS/PNS integrated system.

The hardware structure of the integrated system includes microcontroller units, micro inertial measurement units, bioinspired polarization compass, and GNSS module, etc. Two 32-bit ARM cortex-M7 chips (ATSAMV71Q20) as microcontroller units (MCU) are used to process the polarization information and other navigation information. The STIM300 SINS contains three high accurate advanced micro-electromechanical systems (MEMS) gyroscopes (ADXRS642) and three high-performance accelerometers (ADXL202). The GPS receiver module (NEO-6M) is used to provide the initial position information as well as the “ground truth” to evaluate the positioning performance of the proposed integrated system. The integrated system model is communicated with the computer via a data transceiver (HC-12). The block diagram of the integrated system is shown in Figure 3, and the hardware structure is shown in Figure 4. The parameters of the sensors are listed in Table 1.

## 3. The SINS/PNS Integrated System Modeling

**Figure 5:**
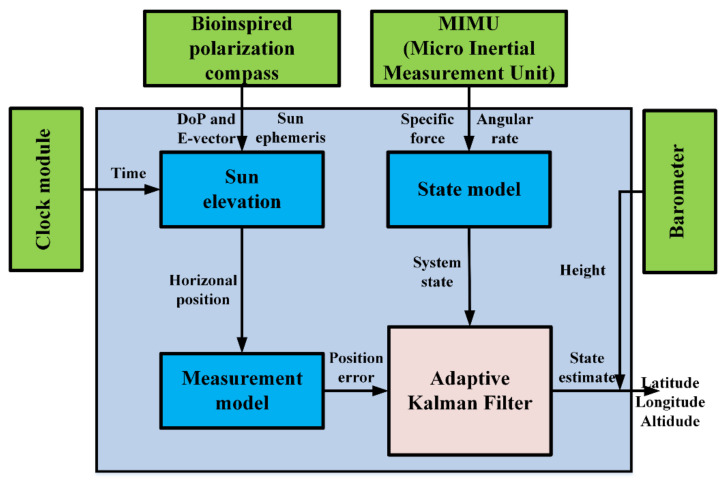
The SINS/PNS integrated system.

In this section, an integrated system model combining the inertia and polarization modules is established, and the sun elevation calculation method based on DoP and E-vector is introduced. The modules of the SINS/PNS integrated system are shown in Figure 5. The micro-inertial measurement unit (MIMU) can measure the incremental angles vector and incremental velocities vector. The bio-inspired polarization compass can obtain the sun elevation in the b-frame. The clock module and barometer are used to provide the time and height information, respectively.

### 3.1. The State Model of the System

The state model of the SINS errors is used in this paper as follows [24]:

(1) $$\dot{x}(t)=F{\left(t\right)}_{SINS}x(t)+G{\left(t\right)}_{SINS}w{\left(t\right)}_{SINS}$$

where $F{\left(t\right)}_{SINS}$ is the state transition matrix of the SINS; $G{\left(t\right)}_{SINS}$ is the noise matrix of the SINS; $w{\left(t\right)}_{SINS}$ is the white noise of the gyroscopes and accelerometers. The details of the state transition matrix $F{\left(t\right)}_{SINS}$, the system noise matrix $G{\left(t\right)}_{SINS}$, and the white noise matrix $w{\left(t\right)}_{SINS}$ can be found in [24]. In addition, x(t) is the system state vector, which can be expressed as:

(2) $$x(t)={\left[\begin{array}{ccccc} \begin{array}{ccc} \phi_{E} & \phi_{N} & \phi_{U} \end{array} & \begin{array}{ccc} \delta V_{E} & \delta V_{N} & \delta V_{U} \end{array} & \begin{array}{ccc} \delta L & \delta \lambda & \delta h \end{array} & \begin{array}{ccc} \varepsilon_{x} & \varepsilon_{y} & \varepsilon_{z} \end{array} & \begin{array}{ccc} \nabla_{x} & \nabla_{y} & \nabla_{z} \end{array} \end{array}\right]}^{T}$$

where $\phi_{E},\;\phi_{N},\;\mathrm{and}\;\phi_{U}$ denote the east, north, and upwards misalignment angles of SINS; $\delta V_{E},\;\delta V_{N},\;\mathrm{and}\;\delta V_{U}$ are the east, north, and upwards velocity errors of SINS; δL, δλ, and δh are the latitude, longitude, and height position errors of SINS; $\varepsilon_{x},\;\varepsilon_{y},\;\mathrm{and}\;\varepsilon_{z}$ are the constant drifts of gyroscopes in the *x*-axis, *y*-axis, and *z*-axis, respectively. $\nabla_{x},\;\nabla_{y},\;\mathrm{and}\;\nabla_{z}$ are the biases of accelerometers in the *x*-axis, *y*-axis, and *z*-axis, respectively.

### 3.2. The Measurement Model of the System

The traditional position measurement models are based on the position errors, which are calculated by the polarization compass and INS. In this way, the position calculation errors from the heading information and sun azimuth will inevitably be introduced into the integrated system model. Hence, to reduce the position calculation errors caused by the heading sensor and the polarization compass, a position measurement model based on sun elevation is established.

#### 3.2.1. The Sun Elevation Calculation

**Figure 6:**
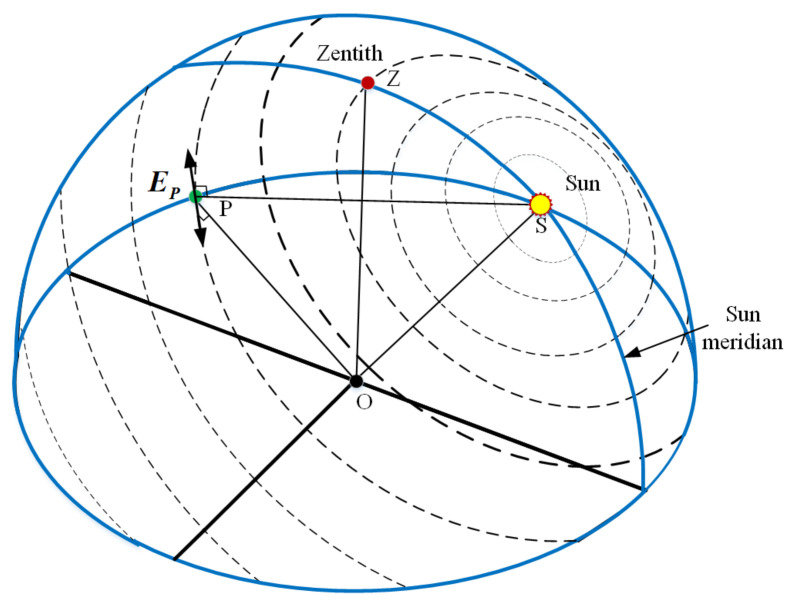
Skylight polarization pattern. The direction and width of the bars represent the E-vector and degree of polarization (DoP) in the sky. The wider the width of the bars, the greater the DoP value, and the DoP reaches a maximum at an angular distance of 90° away from the sun. Point O denotes the position of the observer, and point P is an observed position in the sky. $E_{P}$ is the E-vector of the observed position (P), which is perpendicular to both the observed vector (OP) and the sun vector (OS). The skylight polarization pattern is symmetrical about the sun meridian.

When the sunlight transmits through the atmosphere, the polarized skylight is generated by the atmospheric scattering of air molecules and aerosol particles. The skylight polarization information measured at a certain point consists of the DoP and E-vector. The all-sky polarized light information forms the skylight polarization pattern, as shown in Figure 6, which depends on the sun position and the time. Hence, the sun elevation information can thereby be deduced from the obtained polarization information.

The sun elevation can be calculated based on the DoP and E-vector. By measuring the polarized skylight cues, the two polarization observation points with the highest DoP values are selected for fusion. The detailed procedure of the sun elevation calculation method is summarized in what follows.

The two selected E-vectors with the two largest DoP values are denoted as $E_{i}^{m}$ and $E_{j}^{m}$, which can be expressed as follows:

(3) $$\begin{array}{l} E_{i}^{m}=\Omega_{1}{\left[\begin{array}{ccc} \cos\varphi_{i} & \sin\varphi_{i} & 0 \end{array}\right]}^{T}\\ E_{j}^{m}=\Omega_{2}{\left[\begin{array}{ccc} \cos\varphi_{j} & \sin\varphi_{j} & 0 \end{array}\right]}^{T} \end{array}$$

where the coefficients $\Omega_{1}$ and $\Omega_{2}$ can take the value 1 or −1. In addition, $\varphi_{i}$ and $\varphi_{j}$ are the polarization angles of the ith and jth observation points. The two largest DoP values corresponding to the two E-vectors are denoted as follows:

(4) $$D_{\max}\geq D_{i}\geq D_{j}\geq D_{k},\;(i\neq j\neq k,k=1,2,\cdots n)$$

where $D_{i}$ and $D_{j}$ are the DoP of the ith and jth observation points; n is the number of the observation points; and $D_{\max}$ is the maximum DoP value in the overall sky, which is usually less than 75%.

The E-vectors $E_{i}^{m}$ and $E_{j}^{m}$ in the b-frame can be expressed as follows:

(5) $$E_{i}^{b}=C_{mi}^{b}E_{i}^{m},\;E_{j}^{b}=C_{mj}^{b}E_{j}^{m}$$

where $C_{mi}^{b}$ and $C_{mj}^{b}$ are the attitude transfer matrices between the m-frame and the b-frame of the ith and jth observation points, respectively.

Based on the single-scattering Rayleigh atmosphere model, the E-vector is always perpendicular to the sun vector. Hence, the sun vector in the b-frame ($S_{sun}^{b}$) can be calculated as follows:

(6) $$S_{sun}^{b}=\frac{E_{i}^{b}\times E_{j}^{b}}{\sin\langle E_{i}^{b},E_{j}^{b}\rangle }=\frac{C_{mi}^{b}E_{i}^{m}\times C_{mj}^{b}E_{j}^{m}}{\sin\langle E_{i}^{m},E_{j}^{m}\rangle }\;={\left[\begin{array}{ccc} s_{x}^{b} & s_{y}^{b} & s_{z}^{b} \end{array}\right]}^{T}$$

where $\langle E_{i}^{b},E_{j}^{b}\rangle$ is the angle between $E_{i}^{b}$ and $E_{j}^{b}$; and $\langle E_{i}^{m},E_{j}^{m}\rangle$ is the angle between $E_{i}^{m}$ and $E_{j}^{m}$. The $S_{sun}^{b}$ vector can be deduced by the time and attitude information of the system:

(7) $$\left\{\begin{array}{l} s_{x}^{b}=\cos A_{sun}^{b}\cos H_{sun}^{b}\\ s_{y}^{b}=\sin A_{sun}^{b}\cos H_{sun}^{b}\\ s_{z}^{b}=\sin H_{sun}^{b} \end{array}\right.$$

where $A_{sun}^{b}$ and $H_{sun}^{b}$ are the sun azimuth and sun elevation in the b-frame. Hence, the sun elevation in the b-frame can be represented as:

(8) $$H_{sun}^{b}=\arcsin(s_{z}^{b})$$

Different from the method used in [21], the proposed method in this paper takes into account both the DoP and E-vector information of the polarization sensors to improve the accuracy and environmental adaptability of the sun elevation calculation approach.

#### 3.2.2. Position Measurement Modeling

The sun elevation $H_{sun}^{n}$ in the n-frame can be obtained by transforming the sun elevation $H_{sun}^{b}$ in the b-frame. In addition, according to the relationship between the sun position and the observation position, the sun elevation $H_{sun}^{c}$ calculated by the inertial navigation system can be derived as follows:

(9) $$\sin H_{sun}^{c}=\sin L_{ins}\sin Dec_{sun}+\cos L_{ins}\cos Dec_{sun}\cos LHA_{sun}$$

where

(10) $$LHA_{sun}=\left\{\begin{array}{ll} GHA_{sun}+\lambda_{c} & 0<GHA_{sun}+\lambda_{c}<2\pi \\ GHA_{sun}+\lambda_{c}+2\pi & GHA_{sun}+\lambda_{c}<0\\ GHA_{sun}+\lambda_{c}-2\pi & GHA_{sun}+\lambda_{c}>2\pi \end{array}\right.$$

Here, $L_{ins}$ and $\lambda_{ins}$ are the latitude and longitude provided by the INS; $Dec_{sun}$ is the declination of the sun; $GHA_{sun}$ is the Greenwich hour angle of the sun, which is related to the time; and $LHA_{sun}$ is the local hour angle of the sun, which is related to the time and location.

Based on the position information provided by the INS and the accurate position information, we can get the following relationship:

(11) $$\left\{\begin{array}{l} L_{ins}=L_{real}+\delta L\;\\ \lambda_{ins}=\lambda_{real}+\delta \lambda \\ H_{sun}^{c}=H_{sun}^{n}+\delta H_{sun} \end{array}\right.$$

where $L_{real}$ and $\lambda_{real}$ are the real latitude and longitude, respectively; δL and δλ are the latitude and longitude errors; and $\delta H_{sun}$ is the sun elevation error calculated by the INS.

Assuming that the sun elevation error $\delta H_{sun}$ is a small value, we can get an approximate expression as follows:

(12) $$\left\{\begin{array}{l} \sin(\delta H_{sun})\approx \delta H_{sun}\\ \cos(\delta H_{sun})\approx 1 \end{array}\right.$$

Based on Equations (9), (11), and (12), we can get the following expression by performing the first-order Taylor expansion on the point $L_{real}$ and $\lambda_{real}$:

(13) $$\sin(H_{sun}^{c})=\sin(H_{sun}^{n}+\delta H)=\sin H_{sun}^{n}+\cos H_{sun}^{n}\delta H$$

The system measurement model can also be expressed as follows:

(14) $$z\left(t\right)=\sin H_{sun}^{c}-\sin H_{sun}^{n}=H\left(t\right)x\left(t\right)+v\left(t\right)$$

where v(t) is the measurement noise of the polarization compass; z(t) is the measured value; and $H\left(t\right)$ is the measurement matrix, which can be represented as follows:

(15) $$H\left(t\right)=\left[0_{1\times 3},\;\alpha ,\;\beta ,\;0_{1\times 10}\right]$$

(16) $$\left\{\begin{array}{l} \alpha =-\cos Dec_{sun}\cos L_{ins}\sin LHA_{sun}\\ \beta =\sin Dec_{sun}\cos L_{ins}-\cos Dec_{sun}\sin L_{c}\cos LHA_{sun} \end{array}\right.$$

The Kalman filter (KF) based methods are generally used for the state estimation. However, due to the unknown statistics of the system noises in Equation (1) and the measurement noises in Equation (14), it is difficult to achieve the optimal KF estimation. To solve this problem, the adaptive KF is adopted to estimate the navigation information measured by the polarization compass and SINS in this paper [25]. The parameters of the adaptive Kalman filter are selected according to the sensor’s specifications.

## 4. The Experimental Scheme and Results

### 4.1. The Outdoor Experiment

**Figure 7:**
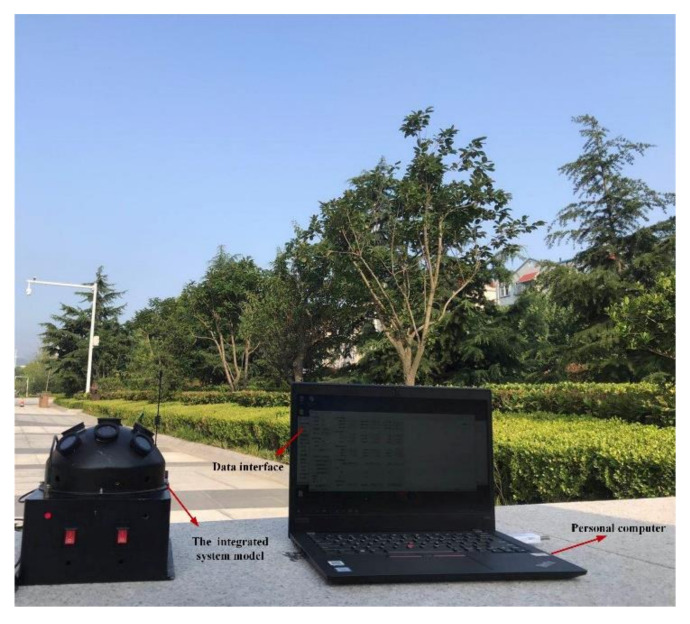
The test equipment in the outdoor environment.

To verify the positioning performance of the proposed integrated system model, an outdoor experiment was carried out under static conditions on 18 September 2019. The test site was in the garden of the liberal arts school (39.979° N, 116.339° E) in Beihang University, Haidian District, Beijing. The positioning test started at 16:14 (Beijing time zone), which lasted 20 min. The test settings are shown in Figure 7. During the test, the sky was clear and cloudless. The sun elevation was lower and the DoP distribution pattern across the sky is significant. The GPS was adopted to provide the initial positioning and also to provide the reference to evaluate the system positioning performance.

### 4.2. The Positioning Performance of the Integrated System Model

**Figure 8:**
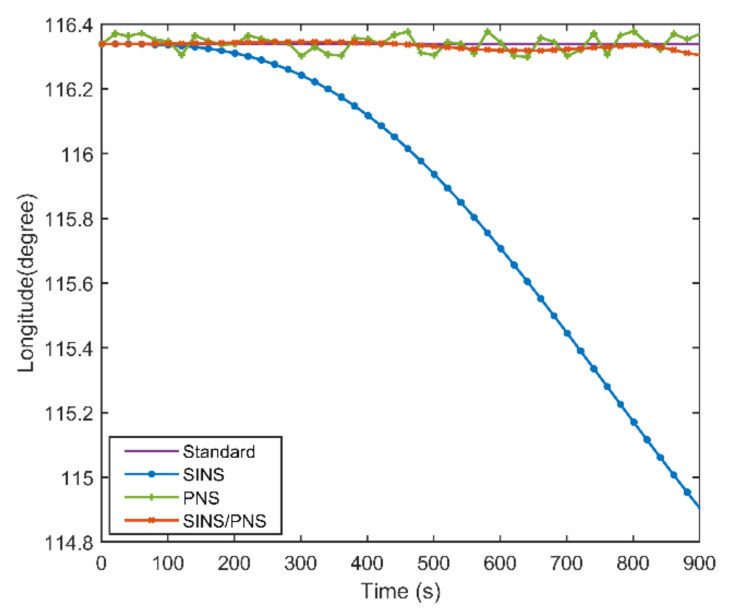
Comparison of the estimated longitude with different models.

**Figure 9:**
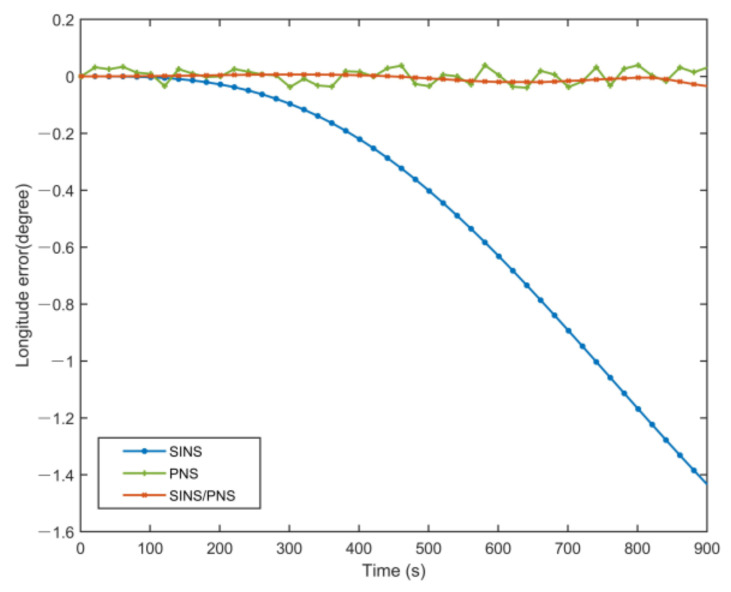
Comparison of the estimated longitude error with different models.

Figure 8 and Figure 9 illustrate the longitude comparison among the SINS/PNS integrated system model (the red curve), the SINS model (the blue curve), and the PNS model (the green curve) [23]. Figure 8 shows that the longitude obtained by the PNS model and the SINS/PNS integrated system model can track the purple reference curve. During the test, the longitude obtained from the proposed model varies from 116.339 to 116.304°, and that obtained from the PNS model varies from 116.378 to 116.299°. The SINS model has the worst performance among the three models. The longitude of the SINS model varies from 116.339 to 114.903°. From Figure 9, we can see that when the test time is less than 200 s, the three models can achieve almost the same longitude calculation accuracy. The longitude errors of the SINS become more significant and have a clear divergent trend after 200 s. The longitude errors of the SINS are larger than 1.4° during the test. Whereas, the longitude errors of the SINS/PNS integrated system model remain stable and maintain a converged state. The longitude errors of the SINS/PNS integrated system model are less than 0.04°. The longitude errors of the PNS model fluctuate continuously, and the errors are larger than the SINS/PNS integrated system model.

**Figure 10:**
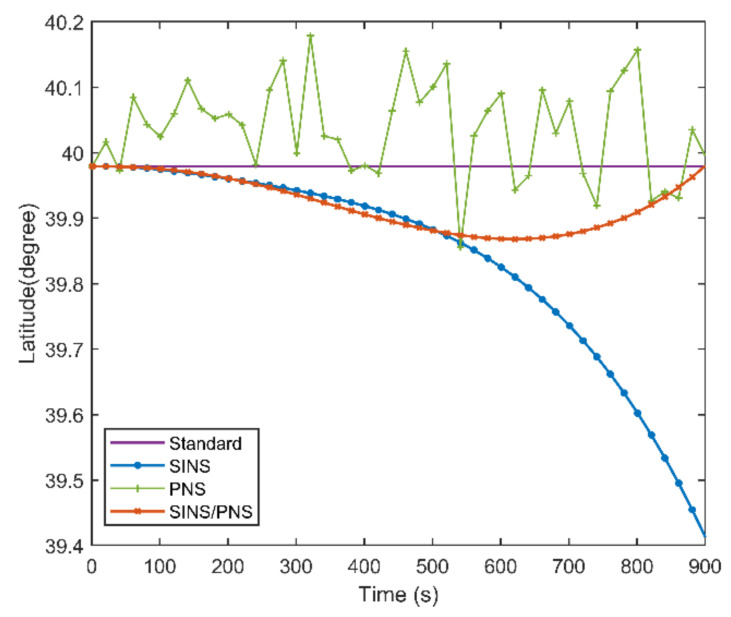
Comparison of the estimated latitude with different models.

**Figure 11:**
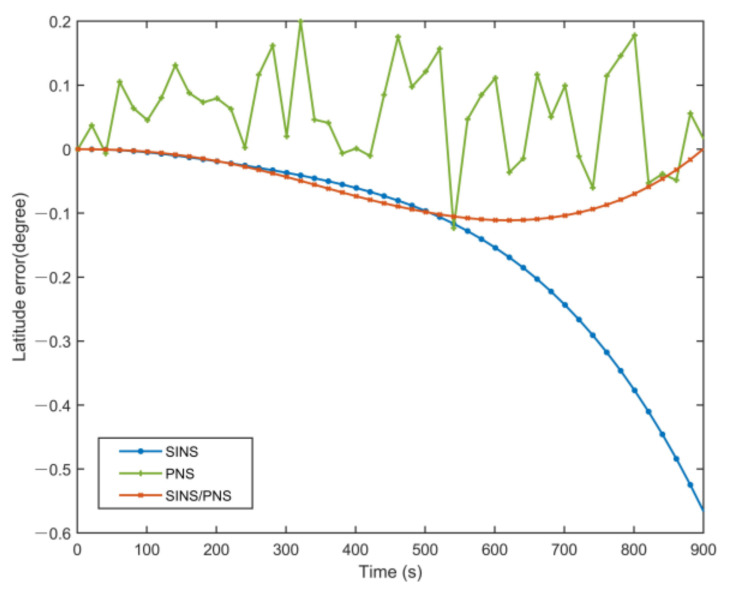
Comparison of the estimated latitude error with different models.

Figure 10 and Figure 11 show the latitude comparison among the SINS/PNS integrated system model (the red curve), the SINS model (the blue curve), and the PNS model (the green curve). From Figure 10, we can see that the latitude calculated based on the SINS model has a clear divergent trend compared with the purple reference curve. The latitude of the SINS model varies from 39.979 to 39.411°. However, the SINS/PNS integrated system model and the PNS model have better performance than the SINS model. During the test, the latitude of the SINS/PNS integrated system model varies from 39.981 to 39.868°, and the PNS model varies from 40.062 to 39.917°. Figure 11 shows that the latitude errors of the SINS increase with time; which vary from 0 to 0.568°. In comparison, the latitude errors of the SINS/PNS integrated system model have a converging trend. The latitude errors of the SINS/PNS integrated system model varies from 0 to 0.034°. The latitude errors of the PNS model fluctuate continuously, and the errors are larger than the SINS/PNS integrated system model.

**Figure 12:**
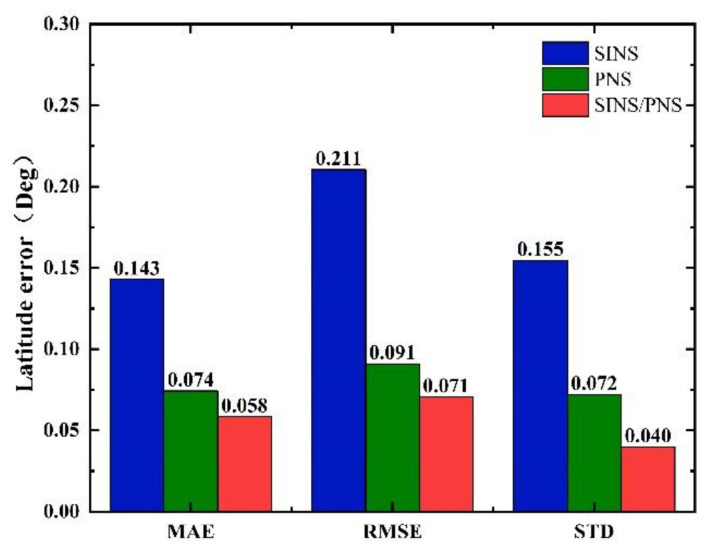
Comparison of statistical characteristics of latitude errors.

**Figure 13:**
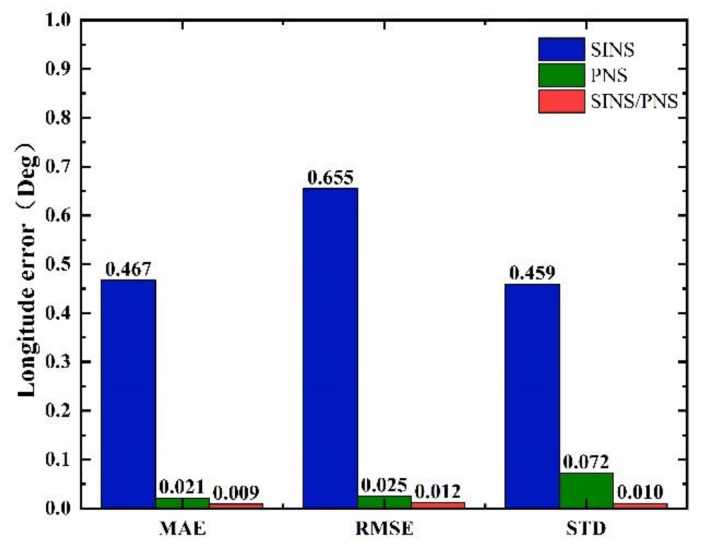
Comparison of statistical characteristics of longitude errors.

The mean absolute error (MAE), root mean square error (RMSE), and standard deviation (STD) of the longitude and latitude of the three models are shown in Figure 12 and Figure 13.

From Figure 12, we can see that the MAE, RMSE, and STD of latitude errors derived from the SINS/PNS integrated system model are 0.058, 0.071, and 0.04°, respectively. Compared to that derived from the SINS model, the errors are reduced by 59.4%, 66.4%, and 74.2%, respectively. Compared to that derived from the PNS model, the errors are reduced by 21.6%, 22.0%, and 44.4%, respectively. Similarly, as can be seen from Figure 13, the MAE, RMSE, and STD of longitude errors derived from the SINS/PNS integrated system model are 0.009, 0.012, and 0.01°, respectively. Compared to that derived from the SINS model, the errors are reduced by 98.1%, 98.2%, and 97.8%, respectively. Compared to that derived from the PNS model, the errors are reduced by 57.1%, 52.0%, and 86.1%, respectively.

From the above results, comparing the three statistical indicators (MAE, RMSE, and STD), we can see that the SINS/PNS integrated system model has the best performance in the position error correction among the three models. More than 97% of improvement in the longitude is achieved compared with the SINS model. The positioning mean absolute error of the proposed model is about 5 km. However, compared with the positioning accuracy of the GPS, the accuracy of the SINS/PNS integrated system model still needs to be improved. The factors that influence the positioning accuracy of the SINS/PNS integrated system model are discussed in the following section.

## 5. Discussion

In this paper, the proposed SINS/PNS integrated system model is based on the sun elevation information. The positioning performance of the model is related to the accuracy of the sun elevation measurement. Although the sun elevation calculation method based on DoP and E-vector can improve the accuracy of the calculated sun elevation, there are still some factors that affect the accuracy of the sun elevation calculation and result in the positioning errors of the integrated system model.

### 5.1. The Installation Errors of the System

**Figure 14:**
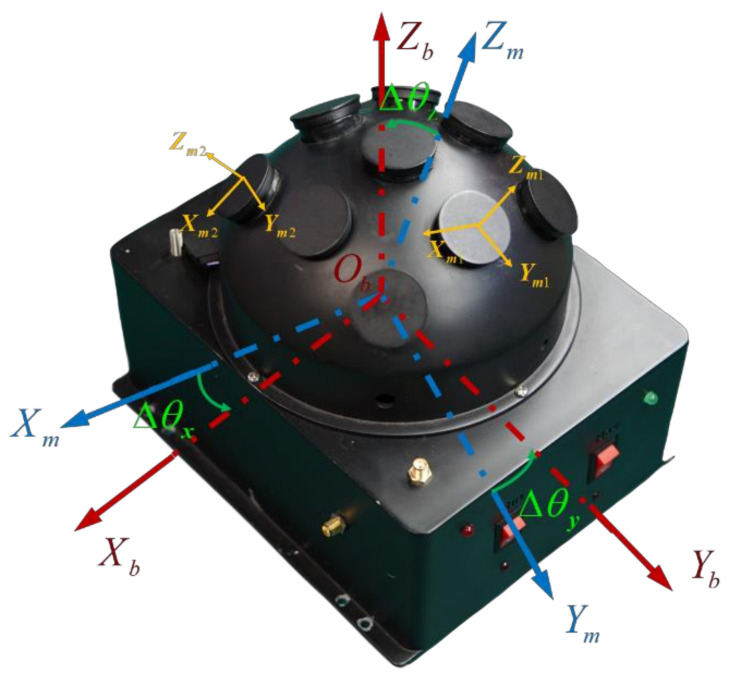
The installation error of the bioinspired polarization compass.

**Figure 15:**
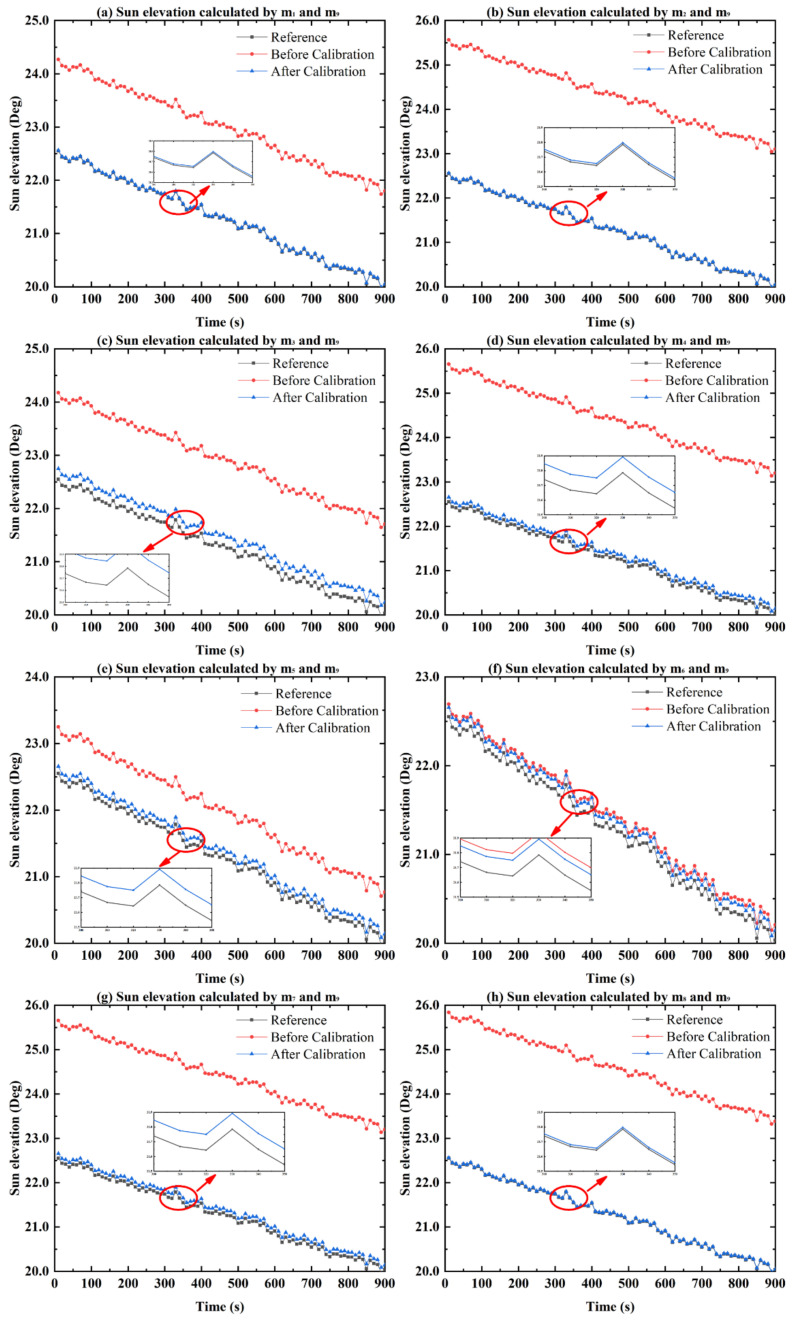
Comparison of the sun elevation before and after calibration. (**a**–**h**) The comparison of the sun elevation before and after calibration calculated by the $m_{1,\;2,3\;\cdots 8}$ and $m_{9}$ polarization sensor units.

The installation errors of the system include the initial installation errors between the bioinspired polarization compass and the b-frame and the initial installation errors among each polarization sensor unit. The installation errors can be described as shown in Figure 14. Under optimal conditions, the m-frame of the bioinspired polarization compass is aligned with the b-frame of the system. However, due to the uncertainty of reference direction and manufacturing defects, the installation errors cannot be avoided. Hence, the E-vectors observed by the polarization sensors are not accurate, and the errors will be passed to the sun elevation calculation. Concerning the installation error analysis and calibration of the system, we have established the calibration model and estimated the installation errors in our works. The calibration results are listed in Table 2. The comparison of the sun elevation before and after calibration is shown in Figure 15.

Figure 15 shows the sun elevation before and after calibration calculated by the $m_{1,\;2,3\;\cdots 8}$ and $m_{9}$ polarization sensor units. It can be seen that after the calibration of in the installation errors, the constant errors of the sun elevation can be compensated. The sun elevation means that the error without calibration (red curve) is 2.11°, which is reduced to 0.08° after calibration. The sun elevation accuracy is improved by 96.2%. It is confirmed that the effect of the installation error of the bioinspired polarization compass can be reduced with the calibration. As a result, the accuracy of the sun elevation can be significantly improved.

### 5.2. The DoP Value and the E-Vector Errors

**Figure 16:**
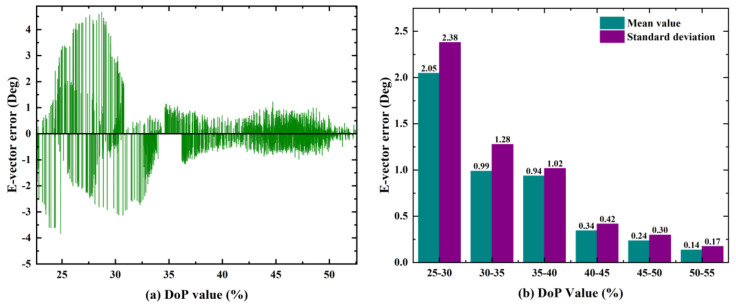
The relationship between the DoP and E-vector error. (**a**) The distribution of the E-vector error in different DoP values; (**b**) the statistical characteristics of the E-vector error in different DoP values.

The bio-inspired polarization navigation is based on the Rayleigh scattering theory, which relies on the single-scattering in clear sky conditions. However, the sky cannot always be clear. Clouds, aerosol, and water are floating in the sky. Due to the influence of environmental interference, the polarization pattern in the sky is destroyed, leading to the deteriorated DoP and E-vector measurements. The calculation accuracy of the sun elevation will be degraded. To illustrate the relationship between the DoP value and the E-vector errors, the outdoor test was carried out. During the test, the sky was cloudless. The results are shown in Figure 16.

From Figure 16a, we can see that the E-vector error gradually increases with the DoP values decreased. When the DoP value is less than 30%, the maximum E-vector error exceeds 4°. For these cases, the E-vector information can be seen as unavailable. Figure 16b shows the statistical results of the DoP and E-vector error. It can be seen that the E-vector accuracy and stability are related to the DoP. When the DoP value is 25%–30%, the mean error of the E-vector is 2.05°, and the standard deviation is 2.38°. When the DoP value is 50%–55%, the mean error of the E-vector is 0.14°, and the standard deviation is 0.17°. The accuracy of the mean value and the standard deviation is improved by 92.7% and 92.8%, respectively.

### 5.3. The Calculation Errors and Model Errors

**Figure 17:**
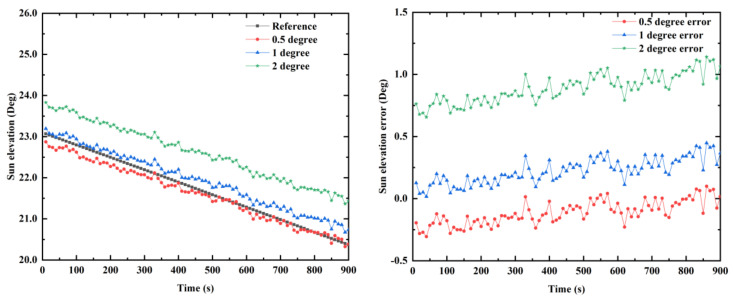
The influence of horizontal attitude errors on the sun elevation calculation accuracy.

To get the sun elevation in the n-frame, the transformation matrix between the n-frame and b-frame must be known. The transformation matrix consists of a horizontal attitude (pitch and roll). The calculation accuracy of the horizontal attitude will affect the accuracy of the sun elevation. Figure 17 illustrates the influence of the horizontal attitude error on the accuracy of the sun elevation calculation. From Figure 17, we can see that as the horizontal attitude error increases, the sun elevation calculation error also increases correspondingly. When the horizontal attitude error is 0.5, 1, and 2°, the mean error of the sun elevation is 0.113, 0.221, and 0.887°, respectively. To reduce the calculation errors of the sun elevation, higher accuracy sensors and algorithms should be used. On the other hand, the proposed system model based on the sun elevation has certain limitations. The sun elevation error calculated by the inertial navigation system is considered a small value. Meanwhile, the statistics of system noises and measurement noises are unknown in the model. Therefore, to improve the accuracy of the model, in future work, it is necessary to further analyze the information on the system noises and measurement noises.

## 6. Conclusions

In this paper, a bionic integrated system combining the polarization compass with inertial sensors is designed. Meanwhile, the SINS/PNS integrated system model based on the sun elevation is proposed. Compared with the existing polarization positioning methods, the positioning method based on the proposed model is autonomous and in real-time. In addition, the sun elevation calculation method based on DoP and E-vector is adopted by the polarization compass to provide the navigation information. The accuracy and environmental adaptability of the sun elevation calculation can be improved. The test results show that the proposed model can reduce the position errors of the SINS with satisfactory performance. Although the positioning accuracy of the proposed model is in a range of few kilometers, the proposed integrated system model is autonomous. It can be used as a backup when the navigation system suffers from electromagnetic interference or GNSS-denied conditions. It can be applied to the vehicle navigation system in a long-endurance and unknown environment.

In addition, the factors that may affect the accuracy of the integrated system model are discussed in the last section. The installation errors of the system can be reduced by calibration. However, there are still some difficulties that we have not solved yet, such as the influence of the external interference (mechanical vibration, attitude error, etc.), the model noises, and different weather conditions, etc. Hence, in the future work, to improve the accuracy of the integrated system model, we will conduct more experiments, analyze the influence of these factors, and utilize the appropriate strategies to reduce their influence.

## Figures and Tables

**Table 1:** Sensor parameters.

Sensors	Parameters
Polarization compass	Angular accuracy: 0.1° (1σ) Output frequency: 1 Hz
Gyroscope	Output frequency: 1 Hz
	Bias stability: 5°/h
	Angular random walk: $0.5º/\sqrt{h}$
Accelerometer	Output frequency: 50 Hz
	Bias stability: 50 ug
	Velocity random walk: $25\;\mu g/\sqrt{\mathrm{Hz}}$
GPS receiver	Output frequency: 50 Hz
	Position accuracy: 2.5 m
	Output frequency: 5 Hz

**Table 2:** Comparison of the E-vector mean error before and after calibration.

The Polarization Sensor Units	The E-Vector Mean Error before Calibration	The E-Vector Mean Error after Calibration
$m_{1}$	−2.9	−0.1
$m_{2}$	−4.3	0.1
$m_{3}$	−2.8	0.3
$m_{4}$	−4.4	0.2
$m_{5}$	−1.8	0.2
$m_{6}$	−1.2	0.2
$m_{7}$	−4.4	−0.2
$m_{8}$	−4.6	−0.1
$m_{9}$	−1.2	−0.1

## Data Availability

Not applicable.
